# Supplementary material for: Protocol for a scoping review of research on abortion in sub-Saharan Africa
Source: PLoS One. 2021 Jul 15;16(7):e0254818. doi: 10.1371/journal.pone.0254818 (PMC8282029; doi:10.1371/journal.pone.0254818)
Supplement: S1 Checklist — (DOCX) [file pone.0254818.s001.docx]

**S1 Checklist. Preferred Reporting Items for Systematic reviews and Meta-Analyses extension for Scoping Reviews (PRISMA-ScR) Checklist**

| **SECTION** | **ITEM** | **PRISMA-ScR CHECKLIST ITEM** | **REPORTED ON PAGE #** |
| --- | --- | --- | --- |
| **TITLE** | | | |
| Title | 1 | **Protocol for a scoping review of research on abortion in sub-Saharan Africa**  . | Title page |
| **ABSTRACT** | | | |
| Structured summary | 2 | Abstract provided | 2 |
| **INTRODUCTION** | | | |
| Rationale | 3 | This review will provide direction for future research, investments, and offer guidance for policy and programming on safe abortion. | 2 |
| Objectives | 4 | i. How has abortion research (e.g., trends in volume, themes, study designs, and African-led studies) evolved in sub-Saharan Africa over the past decade?  ii. What is the geographical landscape of evidence on abortion incidence, the economic burden of unsafe abortion, and the cost and consequences of unsafe abortion to women and girls in sub-Saharan Africa, and what are the key findings? | 6 |
| **METHODS** | | | |
| Protocol and registration | 5 | This submission in lieu of a registration | N/A |
| Eligibility criteria | 6 | We intend to capture all research papers published on abortion in SSA, including those focusing on women, health providers, policymakers, and community members between January 2011 and December 2020. | 7 |
| Information sources* | 7 | We will search the following databases PubMed, HINARI, AJOL, Science Direct, SCOPUS, Web of Science, and CINAHL. | 9 |
| Search | 8 | Search strategy provided | Appendix 1 |
| Selection of sources of evidence† | 9 | Two researchers will independently assess articles for inclusion by screening the titles, abstracts, and full-texts of studies returned through the search process. | 10 |
| Data charting process‡ | 10 | A calibrated form has been developed to capture the requisite data | 12 |
| Data items | 11 | 1. Author 2. Author composition 3. Collaboration types 4. Is there an author from the country of study’s focus? 5. Title 6. Language 7. Country 8. Study setting 9. Sub-region 10. Type of publication 11. Study design 12. Aim/objectives 13. Focus of study 14. Key findings 15. Key Limitations 16. Publication Year 17. Funding 18. Abstract 19. Journal 20. Journal coverage 21. Journal impact factor 22. Link 23. Type of methodology 24. Methodology 25. Theme | 12 |
| Critical appraisal of individual sources of evidence§ | 12 | All indexed sources | Click here to enter text. |
| Synthesis of results | 13 | We will analyze the data using descriptive statistics and thematic analysis, with results organized in tables and charts and presented into themes that reflect the review objectives. Tables will be used to illustrate how abortion research has evolved from January 2011 to July 2021 in terms of volume, themes, study design, African-led papers, and geography | 13 |
| **RESULTS** | | | |
| Selection of sources of evidence | 14 |  | Click here to enter text. |
| Characteristics of sources of evidence | 15 |  | Click here to enter text. |
| Critical appraisal within sources of evidence | 16 |  | Click here to enter text. |
| Results of individual sources of evidence | 17 |  | Click here to enter text. |
| Synthesis of results | 18 |  | Click here to enter text. |
| **DISCUSSION** | | | |
| Summary of evidence | 19 | A summary narrative that synthesizes the information across key themes, including abortion incidence, burden, cost, post-abortion care, and community perception of abortion, will be developed, critically highlighting the advances and gaps in researchers. | 13 |
| Limitations | 20 | This scoping review will only look at research and publications over 10 years (2011-2021), yet obviously, there are equally important articles preceding that period. | 14 |
| Conclusions | 21 |  | Click here to enter text. |
| **FUNDING** | | | |
| Funding | 22 | This research is supported through the generous funding to APHRC by the Swedish International Development Cooperation Agency. | 14 |

JBI = Joanna Briggs Institute; PRISMA-ScR = Preferred Reporting Items for Systematic reviews and Meta-Analyses extension for Scoping Reviews.

* Where *sources of evidence* (see second footnote) are compiled from, such as bibliographic databases, social media platforms, and Web sites.

† A more inclusive/heterogeneous term used to account for the different types of evidence or data sources (e.g., quantitative and/or qualitative research, expert opinion, and policy documents) that may be eligible in a scoping review as opposed to only studies. This is not to be confused with *information sources* (see first footnote).

‡ The frameworks by Arksey and O’Malley (6) and Levac and colleagues (7) and the JBI guidance (4, 5) refer to the process of data extraction in a scoping review as data charting*.*

§ The process of systematically examining research evidence to assess its validity, results, and relevance before using it to inform a decision. This term is used for items 12 and 19 instead of "risk of bias" (which is more applicable to systematic reviews of interventions) to include and acknowledge the various sources of evidence that may be used in a scoping review (e.g., quantitative and/or qualitative research, expert opinion, and policy document).

*From:* Tricco AC, Lillie E, Zarin W, O'Brien KK, Colquhoun H, Levac D, et al. PRISMA Extension for Scoping Reviews (PRISMAScR): Checklist and Explanation. Ann Intern Med. 2018;169:467–473. [doi: 10.7326/M18-0850](http://annals.org/aim/fullarticle/2700389/prisma-extension-scoping-reviews-prisma-scr-checklist-explanation).
