# Supplementary material for: Protocol for a scoping review of research on abortion in sub-Saharan Africa
Source: PLoS One. 2021 Jul 15;16(7):e0254818. doi: 10.1371/journal.pone.0254818 (PMC8282029; doi:10.1371/journal.pone.0254818)
Supplement: S1 Appendix — (DOCX) [file pone.0254818.s002.docx]

# **S1 Appendix. Database Search Strategy**

We will use the advance search builder of the PubMed and HINARI databases, which allows for the use of wildcards (*) and lengthy search terms. We limited our search to a period between 1^st^ January 2011 and 31st July 2021.

Table 1 PubMed Search Strategy

| *#1* | *Abort*ion OR miscarriage OR "Termination of pregnancy" OR "Pregnancy termination"* |
| --- | --- |
| *#2* | *"Abortion, induced"[Mesh] OR "Abortion, threatened"[Mesh] OR "Abortion, Therapeutic"[Mesh] OR "Abortion, Septic"[Mesh] OR "Abortion, Missed"[Mesh] OR "Abortion, Legal"[Mesh] OR "Abortion, Incomplete"[Mesh] OR "Abortion, Habitual"[Mesh] OR "Abortion, Eugenic"[Mesh] OR "Abortion Applicants"[Mesh] OR "Abortion, Criminal"[Mesh] OR "Abortion, Spontaneous"[Mesh]* |
| *#3* | *"post-abortion " OR "post-abortion care"* |
| ***#4*** | ***#1 OR #2 OR #3*** |
| *#5* | *Africa OR "sub-saharan Africa" OR "East Africa" OR "Southern Africa" OR "West Africa" OR "Central Africa"* |
| *#6* | *Angola OR Benin OR Botswana OR "Burkina Faso" OR Burundi OR "Cape Verde" OR Cameroon "Central African Republic" OR Chad OR Comoros OR Congo OR "Democratic Republic of the Congo" OR "Cote d'Ivoire" OR "ivory coast" OR Djibouti OR "Equatorial Guinea" OR Eritrea OR Eswatini OR Swaziland OR Ethiopia OR Gabon OR Gambia OR Ghana OR Guinea OR Guinea-Bissau OR Kenya OR Lesotho OR Liberia OR Madagascar OR Malawi OR Mali OR Mauritania OR Mauritius OR Mozambique OR Namibia OR Niger OR Nigeria OR Rwanda OR "Sao Tome and Principe" OR Senegal OR Seychelles OR "Sierra Leone" OR Somalia OR "South Africa" OR "South Sudan" OR Sudan OR Tanzania OR Togo OR Uganda OR Zambia OR Zimbabwe* |
| ***#7*** | ***#5 OR #6*** |
| ***#8*** | ***#4 AND #7*** |

Table 2 HINARI Search Strategy

| *#1* | *Abort*ion OR miscarriage OR "Termination of pregnancy" OR "Pregnancy termination" OR "Abortion induced" OR "Abortion threatened" OR "Abortion Therapeutic" OR "Abortion Septic" OR "Abortion Missed" OR "Abortion Legal" OR "Abortion Incomplete" OR "Abortion Habitual" OR "Abortion Eugenic" OR "Abortion Applicants" OR "Abortion Criminal" OR "Abortion Spontaneous" OR "post-abortion " OR "post-abortion care"* |
| --- | --- |
| *#2* | *"sub-Saharan Africa" OR "East Africa" OR "Southern Africa" OR "West Africa" OR "Central Africa" OR Angola OR Benin OR Botswana OR "Burkina Faso" OR Burundi OR "Cape Verde" OR Cameron "Central African Republic" OR Chad OR Comoros OR Congo OR "Democratic Republic of the Congo" OR "Cote d'Ivoire" OR "ivory coast" OR Djibouti OR "Equatorial Guinea" OR Eritrea OR Seatini OR Swaziland OR Ethiopia OR Gabon OR Gambia OR Ghana OR Guinea OR Guinea-Bissau OR Kenya OR Lesotho OR Liberia OR Madagascar OR Malawi OR Mali OR Mauritania OR Mauritius OR Mozambique OR Namibia OR Niger OR Nigeria OR Rwanda OR "Sao Tome and Principe" OR Senegal OR Seychelles OR "Sierra Leone" OR Somalia OR "South Africa" OR "South Sudan" OR Sudan OR Tanzania OR Togo OR Uganda OR Zambia OR Zimbabwe* |
| **#3** | **#1 AND #2** |
