# Supplementary material for: Protocol for a scoping review of research on abortion in sub-Saharan Africa
Source: PLoS One. 2021 Jul 15;16(7):e0254818. doi: 10.1371/journal.pone.0254818 (PMC8282029; doi:10.1371/journal.pone.0254818)
Supplement: Attachment — Submitted filename: Response to reviewers commentsAA.docx [file pone.0254818.s004.docx]

Dear Editor,

Please see below our response to all reviewer comments

| **Comment** | **Response** |
| --- | --- |
| 1. Please ensure that your manuscript meets PLOS ONE's style requirements, including those for file naming. The PLOS ONE style templates can be found at <https://journals.plos.org/plosone/s/file?id=wjVg/PLOSOne_formatting_sample_main_body.pdf> and https://journals.plos.org/plosone/s/file?id=ba62/PLOSOne_formatting_sample_title_authors_affiliations.pdf | We have modified the submission in line with PLoS requirements |
| 2. Please include captions for your Supporting Information files at the end of your manuscript, and update any in-text citations to match accordingly. Please see our Supporting Information guidelines for more information: http://journals.plos.org/plosone/s/supporting-information. | Supporting information is now properly captioned |
| Reviewer #1: The protocol is well written. I recommend minor revision before acceptance | Thank you for the positive feedback |
| 1. throughout the manuscript: references should be modified according to style recommended by PLOS journals. | We have modified the referencing and in-text citation to reflect the PLOS journals style |
| 2. Methods • Please explain why you selected French-language articles? | We included French language papers to account for the francophone contributions to abortions research. In addition we have two French speakers in our research team |
| • Why you limited your searches up to December 31, 2020. Why you don’t extend this time for articles published articles in 2021? | We have extended the search period to include July 31 2021 |
| • Web of science is not a database. Do you mean Web of science core collection? | We agree and have removed it from the list |
| **Reviewer #2:** comments can be seen in the attached file |  |
| you can not mention more recently because the information is too old | We have deleted the “More recently” phrase |
| please update all the reports | We have updated all references |
| again this reports are not acceptable as they are too old |  |
| I suggest that this section to be divided into :Research question , Data source and research strategy, citation management, eligibility criteria , title and abstract relevance screening, data characterization, data summary and synthesis. | We are following the PRISMA-P reporting template for scoping reviews 2018 [here](http://www.ncbi.nlm.nih.gov/pubmed/30178033C)  Tricco AC, Lillie E, Zarin W, et al. PRISMA extension for scoping reviews (PRISMA-ScR): checklist and explanation. Ann Intern Med 2018;169:467–73.doi:10.7326/M18- |
